# Development of effective anti-influenza drugs: congeners and conjugates – a review

**DOI:** 10.1186/s12929-019-0567-0

**Published:** 2019-10-23

**Authors:** Jiun-Jie Shie, Jim-Min Fang

**Affiliations:** 1 Institute of Chemistry, Academia Sinica, Taipei, 115 Taiwan; 2 Department of Chemistry, National Taiwan University, Taipei, 106 Taiwan; 3 The Genomics Research Center, Academia Sinica, Taipei, 115 Taiwan

**Keywords:** Influenza, Neuraminidase, Inhibitor, Drug, Congener, Conjugate

## Abstract

Influenza is a long-standing health problem. For treatment of seasonal flu and possible pandemic infections, there is a need to develop new anti-influenza drugs that have good bioavailability against a broad spectrum of influenza viruses, including the resistant strains. Relenza™ (zanamivir), Tamiflu™ (the phosphate salt of oseltamivir), Inavir™ (laninamivir octanoate) and Rapivab™ (peramivir) are four anti-influenza drugs targeting the viral neuraminidases (NAs). However, some problems of these drugs should be resolved, such as oral availability, drug resistance and the induced cytokine storm. Two possible strategies have been applied to tackle these problems by devising congeners and conjugates. In this review, congeners are the related compounds having comparable chemical structures and biological functions, whereas conjugate refers to a compound having two bioactive entities joined by a covalent bond. The rational design of NA inhibitors is based on the mechanism of the enzymatic hydrolysis of the sialic acid (Neu5Ac)-terminated glycoprotein. To improve binding affinity and lipophilicity of the existing NA inhibitors, several methods are utilized, including conversion of carboxylic acid to ester prodrug, conversion of guanidine to acylguanidine, substitution of carboxylic acid with bioisostere, and modification of glycerol side chain. Alternatively, conjugating NA inhibitors with other therapeutic entity provides a synergistic anti-influenza activity; for example, to kill the existing viruses and suppress the cytokines caused by cross-species infection.

## Background

### Influenza is a serious and long-standing health problem

Influenza virus is one of major human pathogens responsible for respiratory diseases, causing high morbidity and mortality through seasonal flu and global pandemics. Vaccines and antiviral drugs can be applied to prevent and treat influenza infection, respectively [1, 2]. Unfortunately, the RNA genome of influenza virus constantly mutates and the genomic segments may undergo reassortment to form new virus subtypes. Although vaccine is the most effective way for prophylaxis of influenza, vaccine formulations must be updated annually due to changes in circulating influenza viruses [3], and the production of influenza vaccine takes several months. If prediction of the incoming influenza strains is incorrect, the vaccines may just give limited efficacy in protection.

**Fig. 1:**
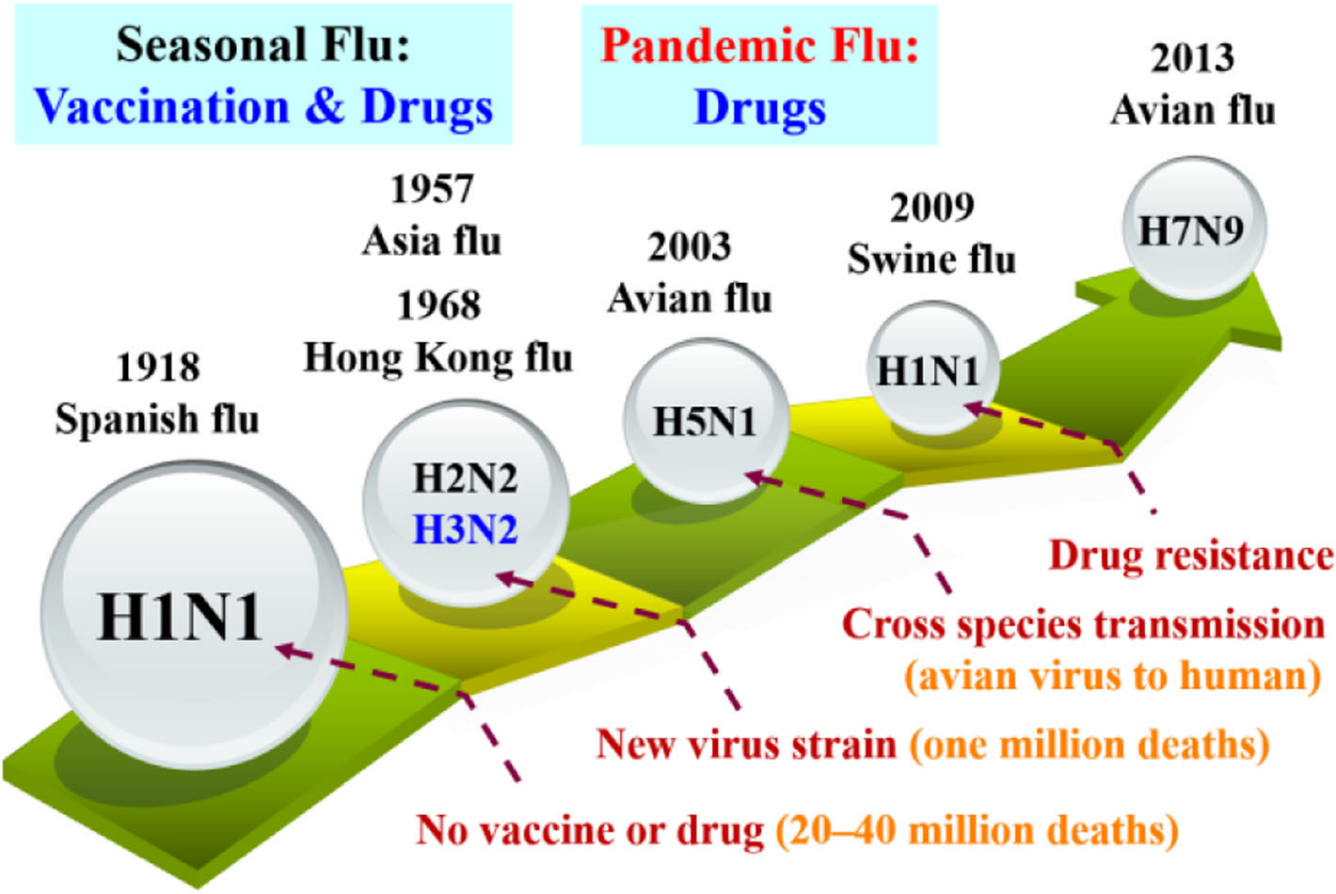
Timeline showing influenza pandemics caused by influenza A viruses

Several influenza pandemics have occurred in the past, such as Spanish flu caused by H1N1 virus in 1918, Asian flu by H2N2 virus in 1957, Hong Kong flu by H3N2 virus in 1968, bird flu by H5N1 and H7N9 viruses in 2003 and 2013, respectively, as well as swine flu by H1N1 virus in 2009 (Fig. 1) [4–6]. The influenza pandemics have claimed a large number of human lives and caused enormous economic loss in many countries. A universal vaccine for flu remains elusive.

### Genome organization of influenza A virus

Influenza viruses are negative-sense RNA viruses of the Orthomyxoviridae family [7]. The viral genome is divided into multiple segments and differs in host range and pathogenicity. There are A, B and C types of influenza viruses, and influenza A viruses are the most virulent. Influenza A viruses infect a wide range of avian and mammalian hosts, whereas influenza B viruses infect almost exclusively humans. Much attention has been paid to influenza A viruses because they have brought about pandemic outbreaks. The structure of influenza virus contains three parts: core, envelope and matrix proteins. These proteins are hemagglutinin (HA), neuraminidase (NA), matrix protein 1 (M1), proton channel protein (M2), nucleoprotein (NP), RNA polymerase (PA, PB1 and PB2), non-structural protein 1 (NS1) and nuclear export protein (NEP, NS2). In addition, some proteins (e.g. PB1-F2, PB1-N40 and PA-X) were found in particular strains [8, 9]. Influenza A viruses are further classified by HA and NA subtypes [10]. There are 18 subtypes of HA and 11 subtypes of NA; for example, H1N1 and H3N2 are human influenza viruses, while H5N1 and H7N9 are avian influenza viruses. HA and NA constantly undergo point mutations (antigenic drift) in seasonal flu. Genetic reassortment (antigenic shift) between human and avian viruses may occur to cause pandemics [11, 12].

### Infection and propagation route of influenza virus

**Fig. 2:**
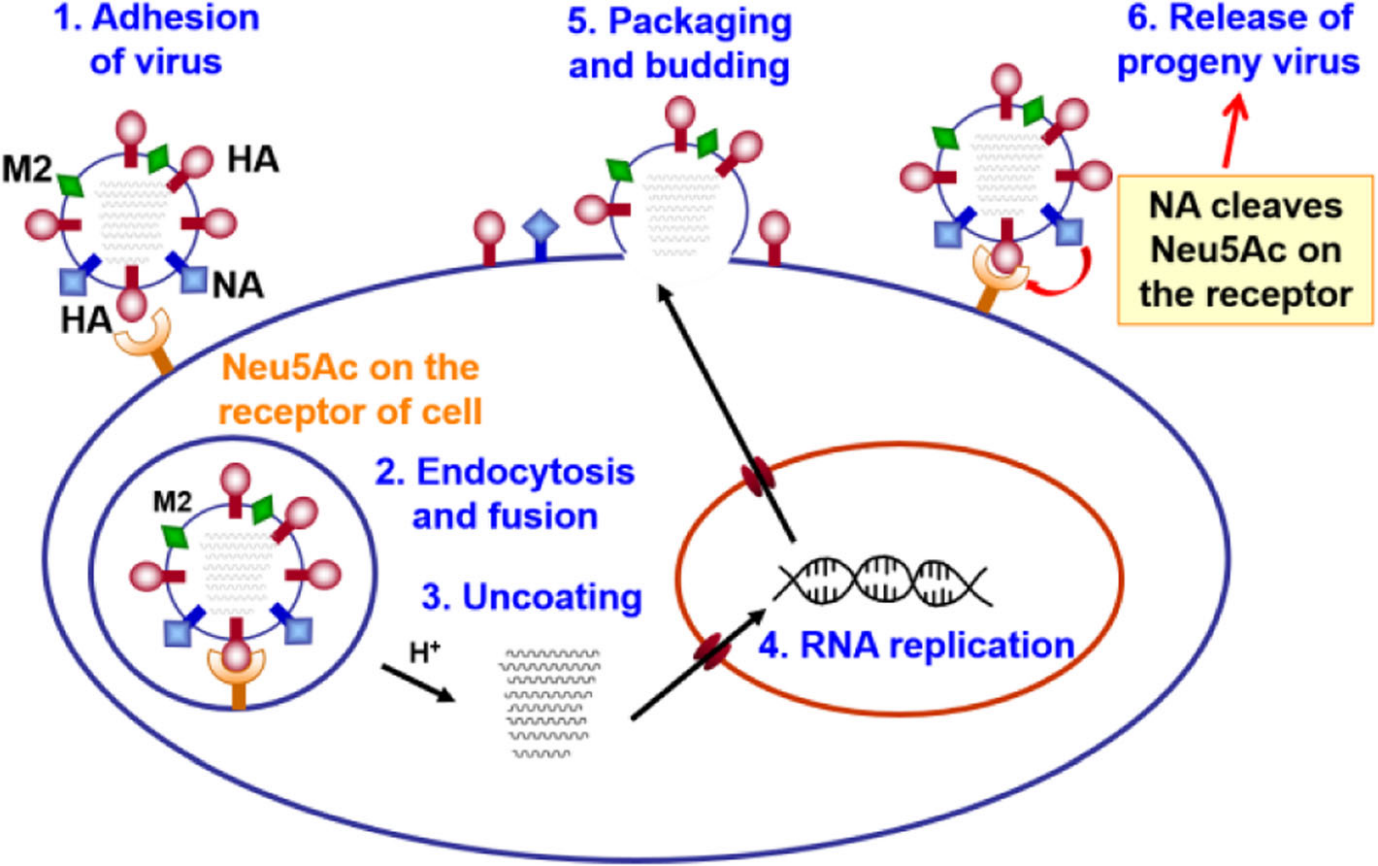
Schematic representation of the life cycle of influenza virus

The life cycle of influenza virus is a complex biological process that can be divided into the following steps (Fig. 2): (i) virion attachment to the cell surface (receptor binding); (ii) internalization of the virus into the cell (endocytosis); (iii) viral ribonucleoprotein (vRNP) decapsidation, cytoplasmic transport and nuclear import; (iv) viral RNA transcription and replication; (v) nuclear exportation and protein synthesis; (vi) viral progeny assembly, budding and release from the cell membrane. All of these steps in the life cycle of influenza virus are essential for its virulence, replication and transmission. Developing a small molecule inhibitor that blocks any of these steps can produce a potentially efficient strategy to control and prevent influenza infection [13].

**Fig. 3:**
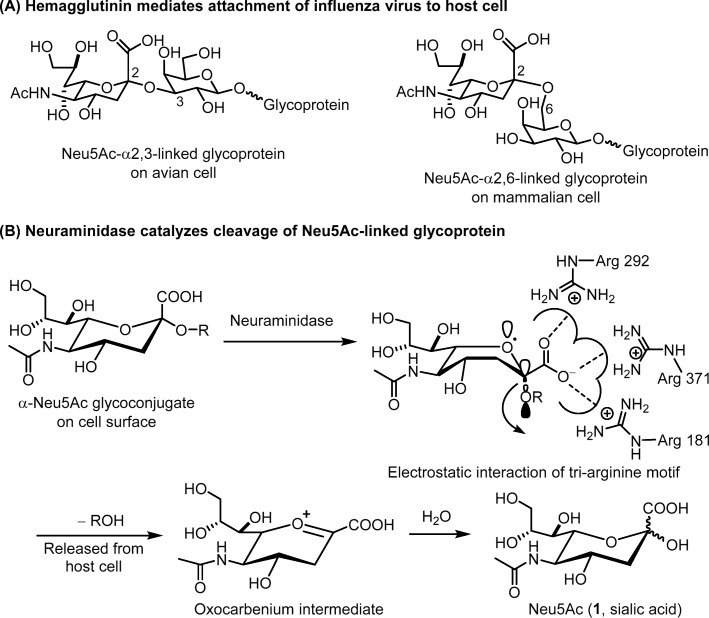
Actions of hemagglutinin and neuraminidase. **a** Binding of HA to the surface Neu5Ac-linked glycoproteins on host cell. **b** NA catalyzes the hydrolytic reaction to cleave the terminal Neu5Ac residue from the sialo-receptor

The influenza HA exists as a trimer and mediates the attachment to host cell via interactions with the cell surface glycoproteins that contain a terminal sialic acid (*N*-acetylneuraminic acid, Neu5Ac, compound **1** in Fig. 3) linked to galactose in α2,3 or α2,6 glycosidic bond [14]. Influenza viruses from avian recognize the 2,3-linked Neu5Ac receptor on host cell, whereas the human-derived viruses recognize 2,6-linked Neu5Ac receptor. The viruses from swine recognize both α2,3 and α2,6 receptors (Fig. 3a). After endocytosis and fusion of the viral envelope membrane into the host endosomal membrane, the viral ribonucleoprotein (RNP) complexes will enter the host cell, and proceed with replication by the machinery of host cell. The newly generated virus will bud on the plasma membrane, and its NA will break the connection between HA and host cell, thereby releasing the progeny virus to infect surrounding cells. NA is a tetrameric transmembrane glycoprotein that catalyzes the hydrolytic reaction to cleave the terminal Neu5Ac residue from the sialo-receptor on the surface of host cell. Thus, HA and NA play the central roles in influenza virus infection [15].

## Development of anti-influenza drugs

Drugs are needed for treatment of patients infected by influenza viruses, especially during influenza pandemics without effective vaccine. Even broadly protective flu vaccines were available, anti-influenza drugs are still needed, especially important for treating the patients with poor responses to vaccination. The currently available anti-influenza drugs directly target the virus at various stages of the viral life cycle, while therapeutics targeting the host are under development [16, 17].

### Approved anti-influenza drugs

**Fig. 4:**
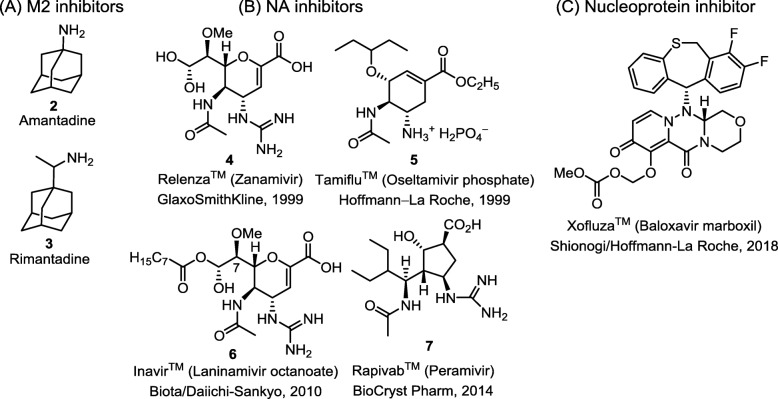
Chemical structures of currently available licensed anti-flu drugs. **a** M2 ion-channel inhibitors, **b** neuraminidase inhibitors, and **c** nucleoprotein inhibitor

Figure 4 shows the approved anti-influenza drugs [18], including M2 ion channel blockers, neuraminidase inhibitors, and a nucleoprotein inhibitor [19]. However, the emerging drug-resistant influenza viruses have posed problems in treatment [20]. Two M2 ion channel inhibitors Fig. 4a (a in black), amantadine (**2**) [21] and rimantadine (**3**) [22], were widely used against influenza. However, the efficacy of M2 ion channel inhibitors is limited to influenza A because influenza B viruses lack M2 protein. In addition, almost all of influenza strains have developed high resistance against both amantadine and rimantadine [23]. The M2 ion channel inhibitors are now largely discontinued and replaced by NA inhibitors [24, 25].

Baloxavir marboxil (Xofluza™, Shionogi/Hoffmann-La Roche, 2018) is used as single-dose oral drug for treatment of influenza [19]. Baloxavir acid, the active form of baloxavir marboxil, is a cap-dependent endonuclease inhibitor targeting the viral PA polymerase and interferes with the transcription of viral mRNA [19]. Moreover, the combination treatment with baloxavir marboxil and oseltamivir, a neuraminidase inhibitor, showed synergistic effect against influenza virus infections in mice experiments [26]. It is possible to develop the combination therapy using sub-optimal dose of baloxavir marboxil and NA inhibitor.

The current medical treatment of influenza patients is based on the administration of neuraminidase inhibitors [27]. NA catalyzes the hydrolytic cleavage of the glycosidic bond of sialic acid, so that the progeny virion can be released from the host cell, and spread to infect the surrounding cells. Thus, an effective way to control influenza is to block the function of NA with specific inhibitors [28]. Currently, four NA inhibitors Fig. 4b are used in clinical practice: zanamivir (**4**) (Relenza™; GlaxoSmithKline, 1999) [29, 30], oseltamivir phosphate salt (**5**) (Tamiflu™; Hoffmann-La Roche, 1999) [31, 32], laninamivir octanoate (**6**) (Inavir™; Biota/Daiichi-Sankyo, 2010) [33] and peramivir (**7**) (Rapivab™; BioCryst Pharm, 2014) [34, 35].

Zanamivir (ZA) is more effective than oseltamivir, but the oral bioavailability of ZA in humans is poor (< 5%) [36], presumably because ZA is a hydrophilic compound that is water soluble and readily eliminated through renal system. ZA is usually delivered by intranasal or dry powder inhalation [29, 30, 37]. After inhaling dry powder, about 7–21% is deposited in the lower respiratory tract, and the rest is deposited in the oropharynx [36]. To prevent influenza, the recommended dose of ZA is 20 mg/50 kg/day for adults by inhalation twice daily (half dose at each inhalation). Adverse drug reactions of zanamivir are rarer than oseltamivir because zanamivir carries a glycerol side chain similar to the chemical structure of sialic acid, the natural NA substrate.

Tamiflu, the phosphate salt of oseltamivir (OS), is a popular orally available anti-flu drug, which is well absorbed and rapidly cleaved by endogenous esterases in the gastrointestinal tract, liver and blood to give OS carboxylate (OC). To treat influenza, the recommended dose of OS for adults is 75 mg, twice a day, for 5 days. Tamiflu is less effective if used after 48 h of influenza infection. The preventive dose is usually 75 mg, once a day for at least 10 days or up to 6 weeks during a community outbreak. In comparison with ZA, oseltamivir has more adverse effects and tends to induce resistant viral strains. The cause of drug resistance is related to the change of binding mode that will be discussed in section 2.3.2.

Laninamivir octanoate is a long-acting anti-flu prodrug that is converted by endogenous esterases in the airway to give laninamivir, the C_7_-methoxy analog of ZA as a potent NA inhibitor [38]. Currently, laninamivir octanoate is only approved for use in Japan to treat and prevent influenza A and B infection. A single inhalation of the drug powder at a dose of 20 mg daily for 2 days is recommended for prophylaxis, and at 40 mg dosage for treatment of individuals greater than or equal to 10 years of age.

Peramivir (PE) has low oral bioavailability and is administered by a single intravenous drip infusion at a dose of 300 mg in 15 min during influenza treatment. PE is a highly effective inhibitor against influenza A and B viruses with good safety. PE can be used to treat the patients who cannot use oral drugs or insensitive to OS and ZA [39].

### Why do we need new anti-influenza drugs?

Anti-influenza drugs are needed to treat seasonal flu and particularly unexpected global influenza infection. Our recent challenge is to deal with new influenza strains, cross-species transmission, and drug resistance. The pandemic influenza A/H1N1 virus in 2009 is currently circulating as a seasonal virus and resistant to M2 inhibitors [40]. Since 2009, only NA inhibitors have been able to provide protection against the circulating human influenza A and B viruses. Small molecular NA inhibitors are powerful tools to fight against influenza viruses. Like other antiviral therapeutics, influenza NA inhibitor is not an exception to encounter the problem of drug-resistant mutations in the target enzyme. Since the drug-resistant H1N1 influenza virus became popular in 2007 and quickly dominated in the 2008–2009 season, the emergence of OS resistance is of particular concern [41, 42]. The resistant phenotype is associated with an H275Y mutation in NA. In comparison with other permissive mutations, H275Y-mutant viruses do not display any fitness deficits, and thus remain in circulation [43, 44]. The clinically relevant H5N1 avian influenza virus from a patient even shows an increasing resistance against OS. Fortunately, the H275Y mutant is still sensitive to ZA.

In this review, we highlight the latest advances in structural modification of oseltamivir, zanamivir and peramivir for the development of effective anti-influenza drugs, especially focusing on using congeners and conjugates of the existing NA inhibitors. Congeners are the related compounds having comparable chemical structures and biological functions, whereas conjugate refers to a compound having two bioactive entities joined by a covalent bond.

### Rational design of neuraminidase inhibitor congeners

#### Mechanism and assay of neuraminidase catalyzed reaction

**Fig. 5:**
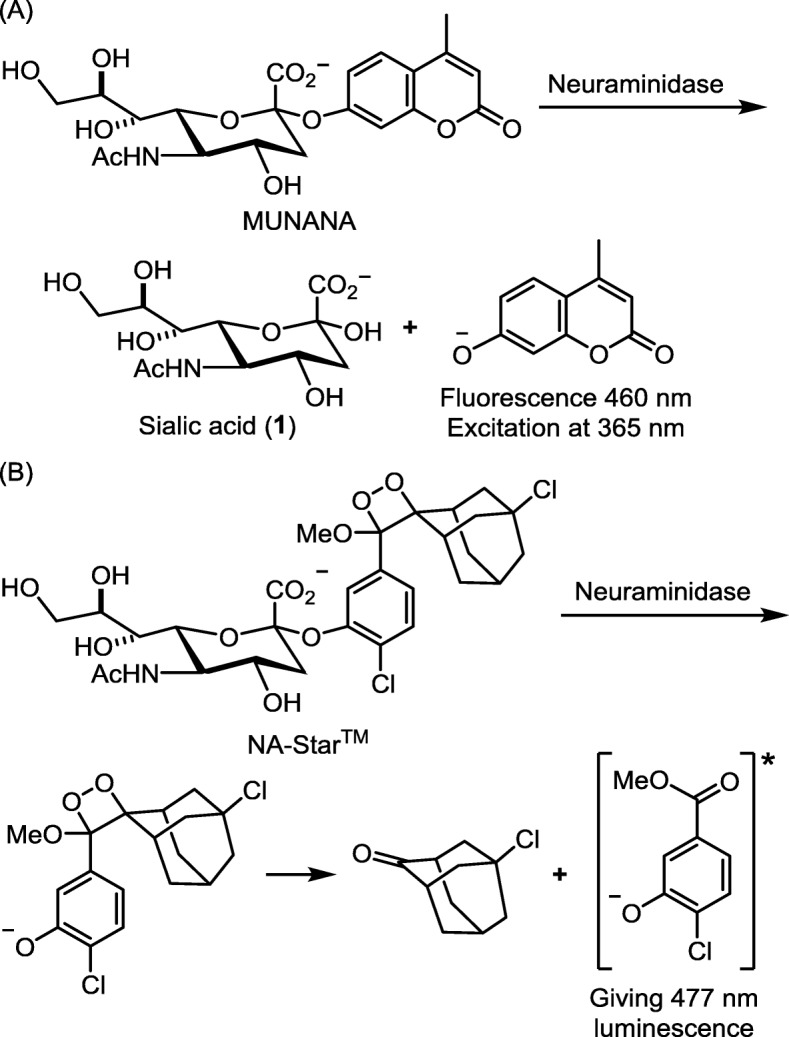
Substrates for assays of influenza NA inhibitors. **a** fluorescent substrate 2-(4-methylumbelliferyl)-α-d-*N*-acetylneuraminic acid (MUNANA), and **b** luminescent substrate NA-Star™

Influenza virus NA is an ideal drug target because NA is an essential enzyme that located on virus membrane for easy access of drugs. Moreover, all subtypes of influenza NAs have a similar conserved active site. On NA-catalyzed hydrolysis of sialo-glycoprotein, the scaffold of Neu5Ac is flipped to a pseudo-boat conformation, so that cleavage of the glycoside bond is facilitated by anomeric effect, giving an oxocarbenium intermediate (Fig. 3b). Based on this reaction mechanism, a fluorometric assay using 2-(4-methylumbelliferyl)-α-d-*N*-acetylneuraminic acid (MUNANA) as NA substrate is designed (Fig. 5a). On hydrolysis of MUNANA, the anion of 4-methylumbelliferone will be released to show strong fluorescence at 460 nm (excitation at 365 nm). The fluorescence dims in the presence of NA inhibitor to suppress the enzymatic hydrolysis. A sialic acid 1,2-dioxetane derivative (NA-Star™, Applied Biosystems) can be used as the luminescence substrate to assess the NA inhibitory activity when the test compound contains a fluorescent moiety to interfere with the fluorescence assay (Fig. 5b).

#### Neuraminidase inhibitors and binding modes

**Fig. 6:**
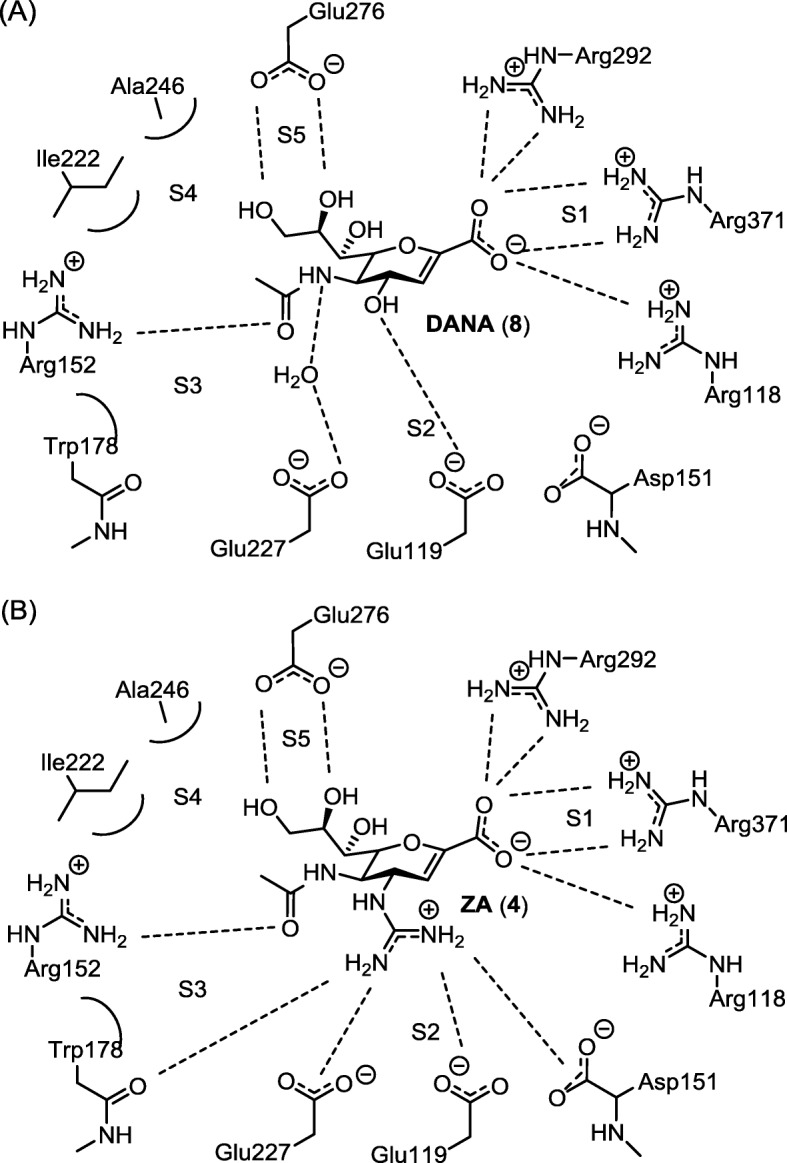
Key interactions of NA inhibitors in the active site based on the crystal structures of the NA–inhibitor complexes. **a** NA–DANA complex; **b** NA–ZA complex

Didehydro-2-deoxy-*N*-acetylneuraminic acid (Neu5Ac2en, DANA, **8**) is the first reported influenza NA inhibitor [45]. The crystal structure of NA–DANA complex (Fig. 6a) has been used as a template for the discovery of more potent NA inhibitors. ZA and OS are two NA inhibitors having (oxa)cyclohexene ring to mimic the oxocarbenium intermediate (Fig. 3). ZA is a DANA guanidino derivative designed by von Itzstein and coworkers [46, 47]; the key interactions of ZA in NA active site are depicted in Fig. 6b. The carboxylate group shows electrostatic interactions with the three arginine residues (Arg118, Arg292 and Arg371 as a tri-arginine motif) in the S1 site of influenza NA [48, 49], whereas the basic guanidino group exhibits strong electrostatic interactions with the acidic residues of Glu119, Asp151 and Glu227 in the S2 site. In addition, the glycerol side chain provides hydrogen bonds with Glu276 in the S5 site.

Oseltamivir carboxylate (OC) contains an amine group at C_5_-position to interact with the acidic residues (Glu119, Asp151 and Glu227). Instead of glycerol side chain, OC has a 3-pentoxy group at the C-3 position. Upon binding to OC, NA redirects the Glu276 residue to Arg224 to form a larger hydrophobic pocket for incorporation of the 3-pentoxy group [50, 51]. However, the salt bridge between Glu276 and Arg224 in H275Y mutant will collapse by substitution of the histidine with a bulkier tyrosine residue, thus altering the hydrophobic pocket of NA and causing decreased affinity with OC [51, 52]. In contrast, ZA rarely induces resistant viruses because it is structurally similar to the natural substrate Neu5Ac.

#### Conversion of carboxylic acid to ester prodrug for better bioavailability

Lipophilicity is an important factor in the pharmacokinetics behavior of drugs. The partition coefficient (log *P*) of a compound between octanol and water can be taken as a measure of lipophilicity. Compounds with log *P* values between − 1 and 5 are likely developed as orally available drugs [53]. In lieu of log *P*, the distribution coefficient (log *D*) between octanol and PBS buffer is used to predict the lipophilicity of ionic compounds.

OC has low lipophilicity and oral bioavailability (< 5%). To solve this problem, the ethyl ester OS was prepared as prodrug with improved oral bioavailability (35%) [54]. The phosphate salt of OS was formulated with appropriate filler materials to make tamiflu capsule with good bioavailability (79%).

A similar strategy has been applied to modify ZA molecule to develop better anti-influenza drugs with improved pharmacokinetic properties and oral bioavailability. Li and coworkers have shown that (heptadecyloxy)ethyl ester of ZA is an effective drug for mice by oral or intraperitoneal administration [55]. Similar to oseltamivir, the ZA ester can undergo enzymatic hydrolysis to release ZA as an active anti-influenza agent. Compared to the rapid elimination of ZA in body, the ZA ester appears to sustain by oral administration. However, no pharmacokinetics studies were performed to determine the value of bioavailability. Amidon and coworkers have synthesized several acyloxy ester prodrugs of zanamivir with conjugation of amino acids [56]. For example, [(L-valyl)oxy] ethyl ester of ZA improved the cell permeability by targeting hPepT1, an oligopeptide transporter present in gastrointestinal tract with broad substrate specificity. This ZA ester is a carrier-linked prodrug with a bioreversible covalent bond, and may be developed as an oral drug.

**Fig. 7:**
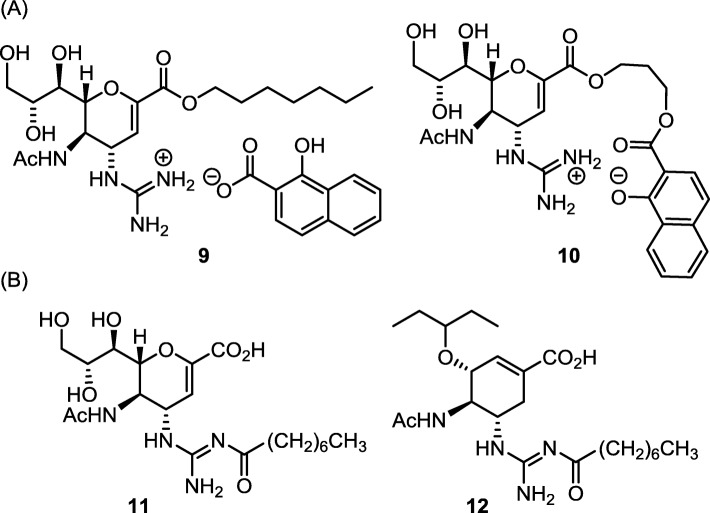
Tackling the hydrophilic guanidinium group in zanamivir and guanidine-oseltamivir carboxylate. **a** Using 1-hydroxy-2-naphthoic acid to form ion-pair. **b** Forming acylguanidine as prodrug

Besides the carboxylate group, the highly hydrophilic guanidinium group also accounts for the low oral bioavailability of ZA and guanidino-oseltamivir carboxylate (GOC). In one approach to improve bioavailability, Amidon and coworkers [57] prepared ZA heptyl ester and used 1-hydroxy-2-naphthoic acid (HNAP) as a counterion of the guanidinium group (Fig. 7a) [58, 59]. This intact ion-pair prodrug (**9**) showed an enhanced permeability across Caco-2 and rat jejunum cell membranes. Moreover, Fang and coworkers have synthesized an intramolecular ion-pair ZA ester prodrug **10** by annexing an HNAP moiety [60]. Compound **10** has improved lipophilicity (log *D* = 0.75 at pH 7.4) by incorporating an aromatic moiety of HNAP and forming the guanidinium–phenoxide ion-pair. The ZA–HNAP prodrug resumes high anti-influenza activity, EC_50_ = 48 nM in cell-based anti-influenza assays, by enzymatic hydrolysis to release zanamivir along with nontoxic HNAP.

#### Conversion of guanidine to acylguanidine for better bioavailability

Though the guanidinium moiety in ZA and GOC plays an important role in NA binding, its polar cationic nature is detrimental to oral administration. Modification of the guanidine group to acylguanidine by attachment of lipophilic acyl substituent improves bioavailability (Fig. 7b) [61]. Moreover, appropriate acyl substituents at the external *N*-position of the guanidine group in ZA are proposed to attain extra bindings in the 150-cavity [47, 62] and 430-cavity [63] of H1N1 virus [61, 64, 65]. Some GOC acylguanidines also possess higher activities than OC against wild-type H1N1 and OS-resistant H259Y viruses [66]. The ZA and GOC acylguanidine derivatives **11** and **12** are stable in acidic media, but slowly hydrolyzed in neural phosphate buffer, and the hydrolytic degradation is accelerated in basic conditions [61]. The hydrolysis of ZA and GOC acylguanidines in animal plasma at physiological condition liberates the parental anti-influenza agents ZA and GOC. Thus, influenza infected mice receiving the octanoylguanidine derivative **11** (or **12**) by intranasal instillation have better or equal survival rate than those treated with parental ZA or GOC [61].

#### Substitution of carboxylic acid with bioisosteres

Bioisosteres are the surrogates mimicking the structure of an active compound while keep similar chemical, physical, electronic, conformational and biological properties [67, 68]. There are two types of bioisosteres, mimicking the enzyme substrate or the reaction transition state. For example, hydroxamic acid, sulfinic acid and boronic acid can mimic the planar structure of carboxylic acid, whereas phosphonic acid, sulfonic acid, sulfonamide, and trifluoroborate can mimic the transition state in enzymatic hydrolysis of peptide bond.

**Fig. 8:**
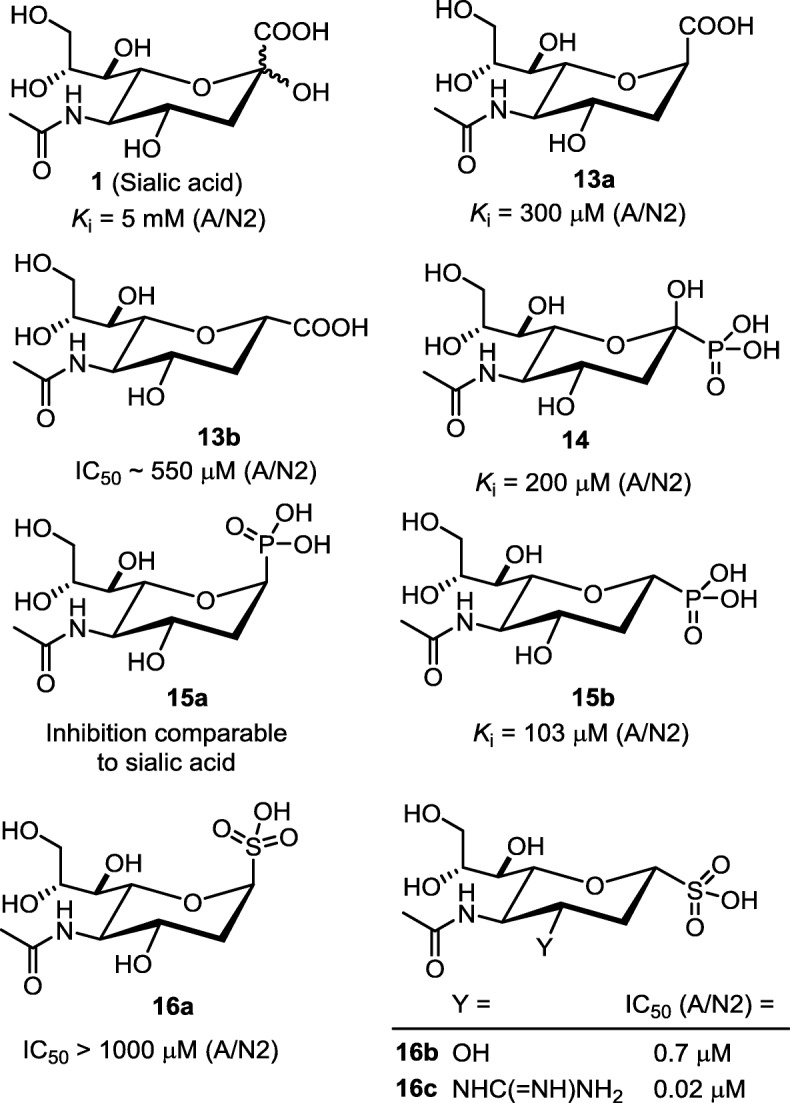
Influenza virus NA inhibitors based on bioisostere-substituted surrogates of sialic acid

Sialic acid (Neu5Ac, **1**), the product of NA-catalyzed hydrolysis, exists as a mixture of two anomers. The affinity of Neu5Ac to influenza NA was weak (*K*_i_ = 5 mM to A/H2N2 virus) [69], presumably due to low proportion (~ 5%) of appropriate anomer in the solution [70]. By substitution of the C_2_-OH group in Neu5Ac with hydrogen atom, the configurations at C-1 position are fixed [71]. Compounds **13a** and **13b** (Fig. 8) have the carboxylate group axially and equatorially located on the chair conformation of pyranose ring, respectively. The inhibition constant of **13b** against *V. cholera* NA is 2.6 mM, but **13a** is inactive.

Considering phosphonic acid and sulfonic acid are more acidic than carboxylic acid, the phosphonate and sulfonate congeners are predicted to have higher affinity toward NA by enhancing the binding strength with the tri-arginine cluster in NA. The phosphonate congener **14** (equatorial PO_3_H_2_) was found to inhibit the NAs of influenza A/N2 and *V. cholera* viruses with IC_50_ values of 0.2 and 0.5 mM, better than the natural carboxylate substrate Neu5Ac [72]. The 2-deoxy phosphonate congeners **15a** (axial PO_3_H) and **15b** (equatorial PO_3_H) were synthesized [71], and shown to bind *V. cholera* NA with *K*_i_ values of 0.23 and 0.055 mM, respectively. In a related study [73], **15b** shows inhibitory activity against H2N2 virus with *K*_i_ and IC_50_ values of 103 and 368 μM, respectively. However, the binding affinity of epimer **15a** is too low to be detected.

The sulfonate derivative **16b** (equatorial SO_3_H) is a more potent inhibitor (*K*_i_ = 2.47 μM against H2N2 virus NA) than the epimer **16a** (axial SO_3_H) and the phosphonate congener **15b** (equatorial PO_3_H) by 14 and 42 fold, respectively. Sulfonate **16b** also inhibits the NAs of H5N1 and the drug-resistant H275Y mutant at the same level with *K*_i_ values of 1.62 and 2.07 μM. In another report [74], the sulfonate derivatives **16a** and **16b** were evaluated for their inhibitory ability against H3N2 (A/Perth/16/2009) virus by fluorometric enzymatic assay. The experiments indicate that **16b** is a much stronger NA inhibitor than the axially substituted sulfonate **16a** (IC_50_ > 1000 μM). The cell-based assay confirms that **16b** has good ability to block H3N2 virus infection of MDCK cells in vitro (IC_50_ = 0.7 μM).

Furthermore, the C_4_-OH group in **16b** is replaced by basic guanidino group to give the derivative **16c** to engage strong bindings with the negatively charged residues (Glu119 and Asp151) in the active site of influenza NA [75]. Thus, the inhibitory activity of **16c** (IC_50_ = 19.9 nM) against H3N2 virus NA is greatly enhanced. The C_3_-guanidino sulfonate **16c** is a very potent inhibitor against influenza NAs of various strains, including H1N1, pandemic California/2009 H1N1 and H5N1-H274Y viruses, with potencies of 7.9 to 65.2 nM. Importantly, **16c** at 1 mM is still inactive to human sialidase Neu2. As **16c** inhibits in vitro infection of influenza H3N2 virus to MDCK-II cells with a high potency of 5 nM, it provides good opportunity for lead optimization.

#### Zanamivir phosphonate congener

**Fig. 9:**
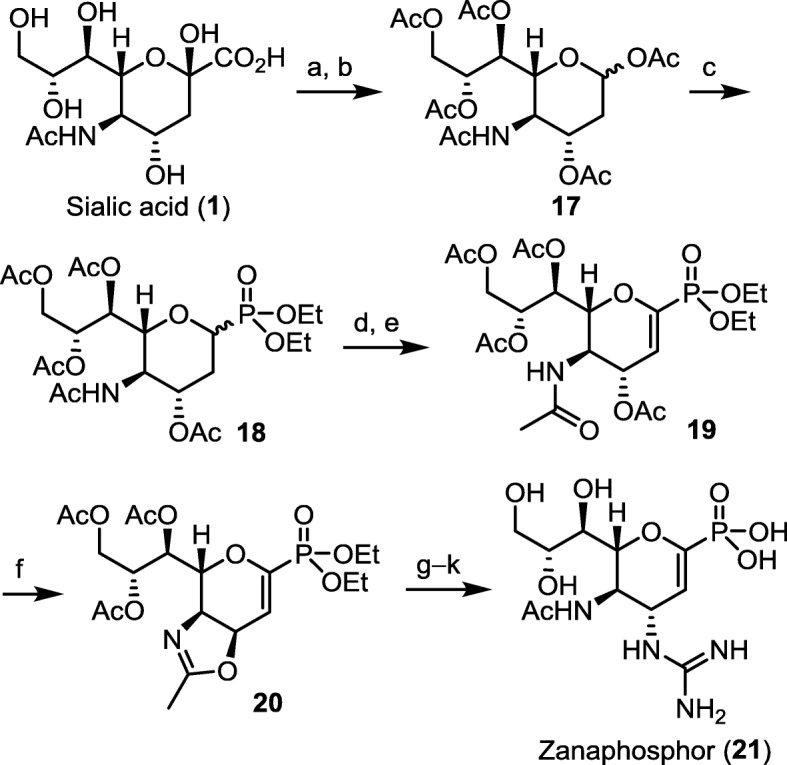
A practical synthesis of zanaphosphor. (a) Ac_2_O, py, rt., 12 h; (b) 100 °C, 5 h, 50% yield for two steps; (c) TMSOTf, P(OEt)_2_OTMS, 0 °C to rt., 24 h, 62% yield; (d) NBS, CH_2_Cl_2_, hv; (e) py, 50 °C, 1 h, 75% yield for two steps; (f) conc. H_2_SO_4_, Ac_2_O, AcOH, rt., 48 h; 80% yield; (g) TMSN_3_; (h) H_2_, Lindlar cat.; (i) MeS-C(=NBoc)NHBoc, HgCl_2_, Et_3_N, CH_2_Cl_2_; (j) TMSBr, CH_2_Cl_2_; (k) MeONa, MeOH, 55% yield for 5 steps. Boc = *tert*-butoxycarbonyl, NBS = *N*-bromosuccinimide, py = pyridine, TMS = trimethylsilyl, TMSOTf = trimethylsilyl trifluoromethanesulfonate

Phosphonate group is commonly used as a bioisostere of carboxylate in drug design [76]. Compared with carboxylic acid (p*K*_a_ = 4.74), phosphonic acid (p*K*_a1_ = 2.38) has higher acidity and stronger electrostatic interactions with guanidinium group. In a helical protein, the formation of phosphonate–guanidinium complex (ΔG^0^ = − 2.38 kJ/mol) is more stable than the carboxylate–guanidinium ion-pair (ΔG^0^ = + 2.51 kJ/mol) [77, 78]. A phosphonate ion in tetrahedral structure is also topologically complementary to bind with Arg118, Arg292 and Arg371 in influenza NAs. The molecular docking experiment [79] shows that zanaphosphor (ZP, compound **21** in Fig. 9), the phosphonate bioisostere of ZA, has higher affinity to NA. Compared the bonding mode of ZA in NA, ZP attains two more hydrogen bonds with the tri-arginine motif while other functional groups (C_4_-guanidinium, C_5_-acetamide and glycerol side chain) maintain comparable interactions. ZP possesses high affinity to influenza NAs with IC_50_ values in nanomolar range. Though the phosphonate analogs (e.g. **14** and **15b**) of sialic acid are weak NA inhibitors with IC_50_ values in sub-millimolar range [72, 80], ZP mimicking the transition state of oxonium-like geometry in the enzymatic hydrolysis is a very effective NA inhibitor. ZP also showed higher activity than ZA in protecting the canine MDCK cells challenged by various influenza viruses including the resistant H275Y strain [79].

The first practical synthesis of ZP was achieved by Fang and coworkers using sialic acid as a viable starting material (Fig. 9) [79]. Sialic acid is firstly protected as a peracetate derivative, which undergoes a concomitant decarboxylation at 100 °C to give the acetyl glycoside **17**. The anomeric acetate was replaced with phosphonate group by using diethyl (trimethylsilyl)phosphite as the nucleophile in the presence of trimethylsilyl trifluoromethanesulfonate (TMSOTf) as a promoter. After photochemical bromination, the intermediate is treated with a base to eliminate an HBr molecule for construction of the oxacyclohexene core structure. Following the previously reported procedure [81], the guanidine substituent is introduced to the C-4 position to furnish ZP. Another synthetic route to ZP is also explored by using inexpensive d-glucono-δ-lactone as the starting material, which proceeds through an asymmetric aza-Henry reaction as a key step [82].

#### Oseltamivir phosphonate congener

**Fig. 10:**
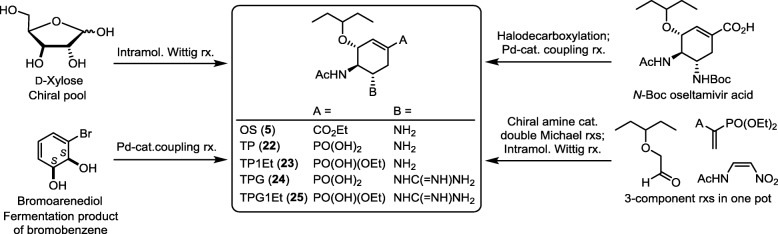
Strategies for synthesis of oseltamivir (OS, **5**), tamiphosphor (TP, **22**), tamiphosphor monoethyl ester (TP1Et, **23**), guanidino tamiphosphor (TPG, **24**) and guanidino tamiphosphor monoethyl ester (TPG1Et, **25**)

In the related study, tamiphosphor (TP, **22**) was synthesized as the phosphonate congener of oseltamivir carboxylate by several methods (Fig. 10). The first synthesis [83] begins with introduction of a (diphosphoryl)methyl substituent to the C-5 position of d-xylose, and the subsequent intramolecular Horner−Wadsworth−Emmons (HWE) reaction constructs the cyclohexene-phosphonate core structure. Intramolecular HWE reaction was also applied to build up the scaffold of the polysubstituted cyclohexene ring in another TP synthesis starting with *N*-acetyl-d-glucosamine (d-GlcNAc) [84]. d-GlcNAc contains a preset acetamido group to manipulate the required absolute configuration in the TP synthesis. In the three-component one-pot approach [85], a chiral amine-promoted Michael reaction of 2-ethylbutanal with nitroenamide, a second Michael addition to 1,1-diphosphorylethene and an intramolecular HWE reaction are sequentially performed in one flask to construct the cyclohexene-phosphonate core structure. TP is thus synthesized by subsequent reduction of the nitro group and hydrolysis of the phosphonate ester. In another synthetic strategy of TP, palladium-catalyzed phosphonylation of 1-halocyclohexene is effectively applied as a key reaction [86–88].

In addition to TP having C_5_-amino substituent, the TPG analog (**24**) having C_5_-guanidino group is also synthesized for evaluation its NA inhibitory activity. It is noted that treatment of phosphonate diethyl esters with bromotrimethylsilane (TMSBr) gives the phosphonic acids TP and TPG, whereas treatment with sodium ethoxide gives the corresponding phosphonate monoesters **23** and **25**.

TP containing a phosphonate group is a potent inhibitor against human and avian influenza viruses, including A/H1N1 (wild-type and H275Y mutant), A/H5N1, A/H3N2 and type B viruses. TPG is even a stronger NA inhibitor because the guanidine group is more basic for stronger interactions with Glu119, Asp151 and Glu227 [18–20, 89].

Though TP (log *D* = − 1.04) has double negative charges on the phosphonate group, it is more lipophilic than OC (log *D* = − 1.69) carrying a single negative charge. The improved lipophilicity of TP is attributable to higher acidity of phosphonic acid to enhance the intramolecular zwitterionic structure or the intermolecular ion-pair structures [57, 60, 90, 91]. The guanidino compounds are also more lipophilic than their corresponding amino compounds because guanidine is more basic and preferable to form zwitterionic/ion-pair structures with the phosphonate group.

Though oseltamivir as a carboxylate ester is inactive to NA, the phosphonate monoester **23** exhibits high NA inhibitory activity because it retains a negative charge in the monoalkyl phosphonate moiety to exert adequate electrostatic interactions with the tri-arginine motif. The phosphonate diester is inactive to NA, while both phosphonate monoesters **23** and **25** show the anti-influenza activity comparable to phosphonic acids **22** and **24**. This result may be attributed to better lipophilicity of monoesters to enhance intracellular uptake. The alkyl substituent in phosphonate monoester can be tuned to improve pharmacokinetic properties including bioavailability. For example, TP and TP monoethyl ester have 7 and 12% oral availability in mice, respectively. It is worth noting that TPG and its monoester **25** also possess significant inhibitory activity against the H275Y oseltamivir-resistant strain with IC_50_ values of 0.4 and 25 nM, respectively. In another study [92], TP monoester molecules are immobilized on gold nanoparticles, which bind strongly and selectively to all seasonal and pandemic influenza viruses through the NAs.

The mice experiments are conducted by oral administration of TP or its derivative after challenge with a lethal dose (10 LD_50_) of influenza virus [93]. When administered at doses of 1 mg/kg/day or higher, TP, TPG and their phosphonate monoesters (**22**–**25**) all render significant protection of mice infected with influenza viruses. Despite the low bioavailability (≤ 12%), all four phosphonates maintain the plasma concentrations in mice above the concentration required to inhibit influenza viruses. The metabolism studies indicate that almost no phosphonate monoesters **23** and **25** were transformed into their parental phosphonic acids **22** and **24**. Therefore, these phosphonate monoesters are active drugs, unlike OS prodrug that releases the active carboxylic acid by endogenous hydrolysis.

#### Peramivir phosphonate congener

**Fig. 11:**
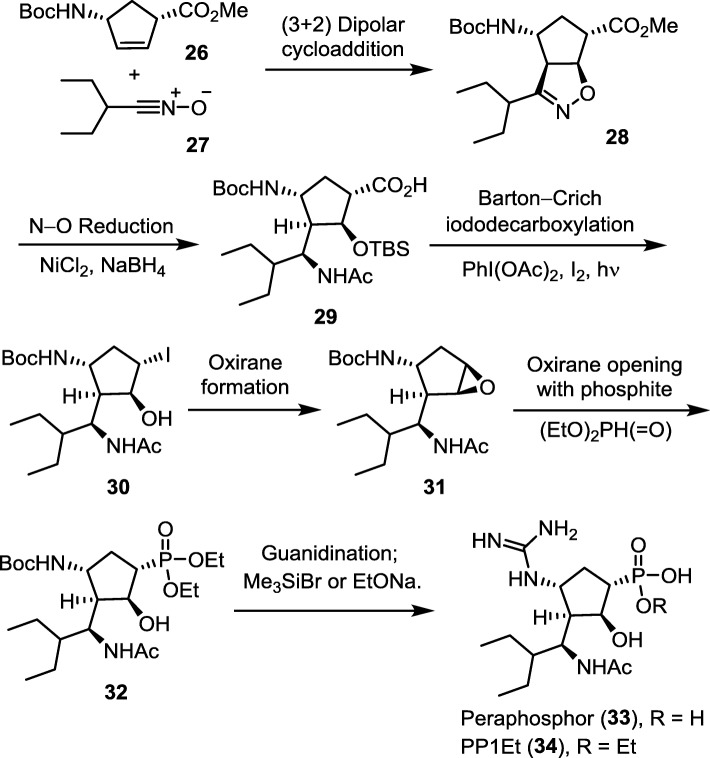
Synthesis of peraphosphor (PP, **33**) and the monoethyl ester (PP1Et, **34**) via a key step of [3 + 2] cycloaddition of 2-ethylbutanenitrile oxide with a cyclopentene dipolarophile

Peraphosphor (PP, **33**) is the phosphonate congener of peramivir (PE). An efficient synthetic method of peraphosphor [94] comprises a [3 + 2] cycloaddition of 2-ethylbuanenitrile oxide (**27**) with a cyclopentene dipolarophile **26** (Fig. 11). After reduction with NiCl_2_ − NaBH_4_ to give multiple substituted cyclopentane-1-carboxylic acid **29**, Barton–Crich iododecarboxylation successfully provides the iodo compound **30** with retention of the *S*-configuration as confirmed by X-ray diffraction analysis. The ring-opening reaction of epoxide **31** is performed at a low temperature (− 78 °C) by using diethyl phosphite and boron trifluoride etherate to afford the phosphonate diester **32**, which is further transformed into PP (**33**) and the phosphonate monoester (**34**).

Although PP is a good NA inhibitor (IC_50_ = 5.2 nM against A/WSN/33 H1N1), its inhibitory activity is unexpectedly 74 times lower than that of PE, contrary to the previous computational study [95] that predicted PP to be a stronger binder for N1 neuraminidase. Due to the flexible cyclopentane core structure, the phosphonate congener (PP) can display different conformation than the carboxylate compound (PE). Therefore, the NA inhibitory activity of PP series is less predictable. The phosphonate compounds **33** and **34** show reduced binding affinity to the H275Y mutant with IC_50_ of 86 and 187 nM, respectively, presumably because less hydrophobic interactions are acquired by the 3-pentyl group in the active site of the mutant NA [96, 97]. However, the phosphonate monoalkyl ester **34** exhibits the anti-influenza activity superior to that of parental phosphonic acid **33** in the cell-based assay. Inferred from the calculated partition and distribution coefficients, the phosphonate monoalkyl ester can increase lipophilicity to enhance intracellular uptake.

Since the crystal structure of PE–NA complex (PDB code: 1L7F) [96] reveals that the C_2_-OH group of peramivir has no direct interaction with influenza NA, a dehydration analog of PP is prepared for bioactivity evaluation. By forming a more rigid cyclopentene ring, the PP dehydration analog regains extensive electrostatic interactions with the tri-arginine cluster in NA, thus exhibiting high NA inhibitory activity (IC_50_ = 0.3 nM) against influenza H1N1 virus.

#### Oseltamivir boronate, trifluoroborate, sulfinate, sulfonate and sulfone congeners

**Fig. 12:**
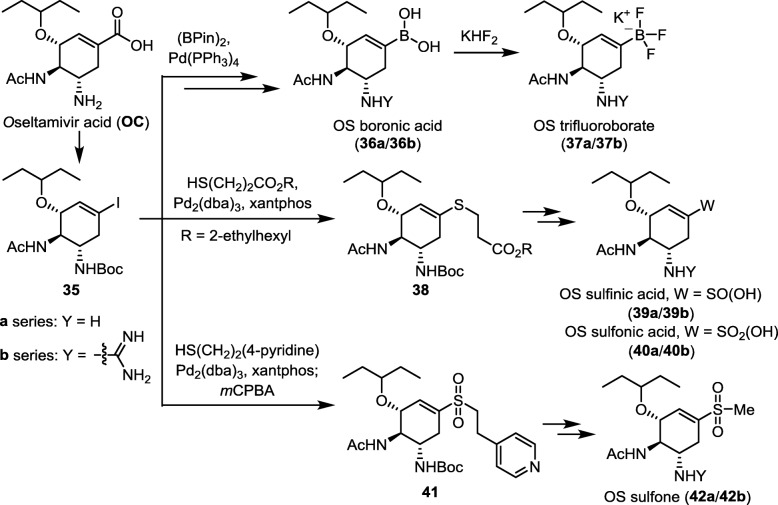
Synthesis of oseltamivir boronates (**36a**/**36b**), trifluoroborates (**37a**/**37b**), sulfinates (**39a**/**39b**), sulfonates (**40a**/**40b**) and sulfones (**42a**/**42b**) from oseltamivir carboxylic acid (OC)

Compared to carboxylic acid (p*K*_a_ ≈ 4.5), boronic acid is a weaker acid (p*K*_a_ ≈ 10.0) while sulfinic acid (p*K*_a_ ≈ 2.0) and sulfonic acid (p*K*_a_ ≈ − 0.5) are stronger acids. Figure 12 outlines the synthetic methods for the oseltamivir boronate, trifluoroborate, sulfinate, sulfonate and sulfone congeners [98]. Oseltamivir carboxylic acid (OC) is converted to a Barton ester, which undergoes photolysis in the presence of CF_3_CH_2_I to give the iodocyclohexene derivative **35**. This pivotal intermediate is subjected to palladium-catalyzed coupling reactions with appropriate diboron and thiol reagents to afford OS boronate (**36a**), trifluoroborate (**37a**), sulfinate (**39a**), sulfonate (**40a**) and sulfone (**42a**) congeners. The corresponding guanidino analogs (GOC congeners) are also synthesized. The GOC congeners (**b** series) consistently display better NA inhibition and anti-influenza activity than the corresponding OC congeners (**a** series). The GOC sulfonate congener (**40b**) is the most potent anti-influenza agent in this series and shows EC_50_ of 2.2 nM against the wild-type H1N1 virus. Since sulfonic acid is a stronger acid than carboxylic acid, it can exert stronger electrostatic interactions than GOC on the three arginine residues (R118, R292 and R371) in the NA active site. The sulfonate compound **40b** may exist in zwitterionic structure and form the sulfonate−guanidinium ion-pair more effectively than GOC to attain higher lipophilicity as predicted by the distribution coefficients (cLog *D*) values. Interestingly, the congeners with trifluoroborate, sulfone or sulfonate ester still exhibit significant NA inhibitory activity, indicating that the polarized B−F and S → O bonds still provide sufficient interactions with the tri-arginine motif.

#### Modification of zanamivir at the glycerol side chain

**Fig. 13:**
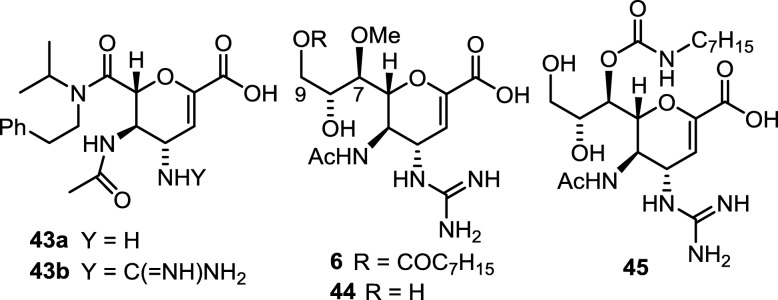
Modification of zanamivir at the glycerol side chain. The C_7_-OH group points away from the NA active site according to the crystallographic analysis of the ZA–NA complex [103]

Replacing the glycerol chain in ZA with tertiary amides (e.g. **43b**, in Fig. 13) still keeps good NA inhibitory activity with the IC_50_ values similar to that of ZA [99, 100]. Compared to the function of 3-pentoxy group in oseltamivir, the dialkylamide moiety in **43b** may render similar hydrophobic interactions in the S5 site of NA. To support this hypothesis, the crystallographic and molecular dynamics studies of compound **43a** with influenza NA were carried out to show that the Glu276 and Arg224 residues form a salt bridge to produce a lipophilic pocket, and an extended lipophilic cleft is formed between Ile222 and Ala246 near the S4 site. The *N*-isopropyl and phenylethyl substituents of **43a** can properly reside in the lipophilic pocket and cleft, respectively [101, 102].

The three-dimensional structure of ZA–NA complex [103] shows that the C_7_-OH group exposes to water without direct interaction with NA. Therefore, the C_7_-OH is an ideal site for structural modification. Laninamivir (compound **44**) derives from ZA by changing the C_7_-OH group to a methoxy group without reduction of NA inhibitory activity. Laninamivir is developed to Inavir (**6**) as a long-acting drug by further converting the C_9_-OH group to an octanoate ester. The lipophilic octanoyl group is proposed to make compound **6** more permeable to cells. Compound **6** is rapidly hydrolyzed by esterases to give laninamivir, which is hydrophilic and may be captured in endoplasmic reticulum and Golgi. When the influenza NA matures in endoplasmic reticulum and Golgi apparatus, laninamivir can firmly retain the NA, thereby preventing the formation of progeny virus particles [104]. The half-life of prodrug **6** was about 2 h in man, and the active ingredient **44** appeared at 4 h after inhalation administration. Compound **44** was slowly eliminated over 144 h [38, 105, 106]. Inavir only needs one inhalation with 40 mg dose to last 5 days for influenza treatment, compared to Relenza and Tamiflu which require twice daily administration at 10 mg and 75 mg doses. Moreover, ZA analogs having the C_7_-OH derived to carbamates (e.g. compound **45**) do not cause significant reduction in anti-influenza activity [107].

### Conjugating neuraminidase inhibitors with enhanced anti-influenza activity

Using NA inhibitor is a good therapy by preventing the spread of progeny viral particles. However, there are related problems in quest of solutions. For example, how to kill the existing viruses in severely infected patients? Is it possible to develop anti-influenza drugs that also suppress the complication of inflammation, especially the cytokine storm caused by cross-species infection? To address these issues, one may consider conjugating NA inhibitors with other therapeutic entity to provide better anti-influenza activity.

Multi-component drug-cocktails may have complex pharmacokinetics and unpredictable drug−drug interactions [108], whereas conjugate inhibitors are designed to incorporate multiple therapeutic entities into a single drug by covalent bond [109, 110].

#### Conjugating zanamivir with porphyrin to kill influenza viruses

Porphyrins and the related compounds have been used as photosensitizers to activate molecular oxygen [111–113]. Activated singlet oxygen (^1^O_2_) is a highly reactive oxidant that can be utilized to kill adjacent cells in photodynamic therapy (PDT), which has been successfully applied to cancer treatment, and occasionally for treatments of bacterial and viral infections [114–116].

**Fig. 14:**
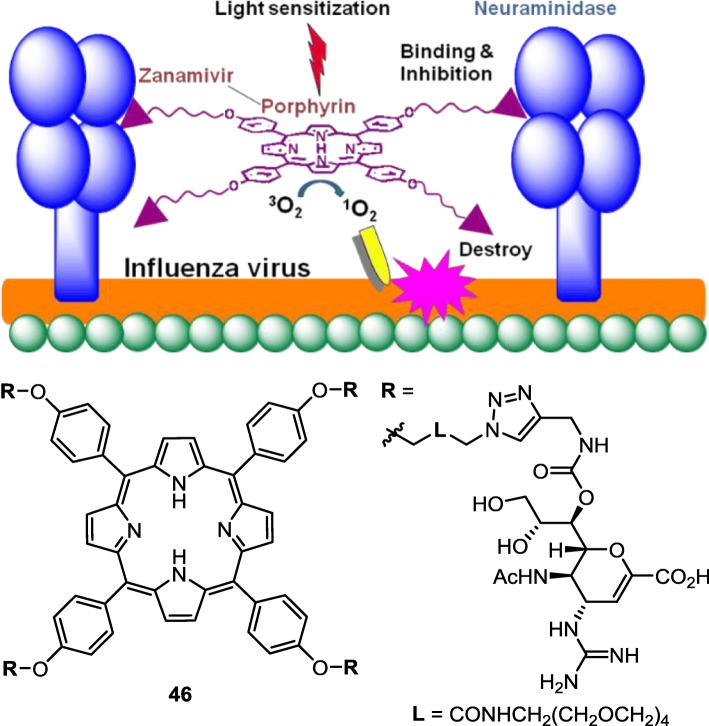
A strategy to kill influenza virus by ZA–porphyrin conjugate. ZA carries the conjugate **46** to viral surface through binding with neuraminidase, and porphyrin is light sensitized (λ_max_ = 420 nm) to generate singlet oxygen in close proximity, causing inactivation of influenza virus

Because ZA has strong affinity to influenza NA, it is an excellent payload to deliver porphyrins to influenza virus in a specific way. Using the C_7_-OH group as connection hinge, four ZA molecules are linked to a porphyrin core structure to furnish the dual functional ZA conjugate **46** (Fig. 14) [117]. The ZA–porphyrin conjugate inhibits human and avian influenza NAs with the IC_50_ values in nanomolar range. By plaque yield reduction assay, conjugate **46** shows 100-fold potency than monomeric ZA in inactivation of influenza viruses. Influenza H1N1 viruses are reduced to less than 5% on treatment with conjugate **46** at 200 nM for 1 h under illumination of room light, whereas 60% titer of viruses remain on treatment with ZA alone or combination of ZA and porphyrin at micromolar concentrations. The viral inactivation by **46** is associated with the high local concentration of the ZA–porphyrin conjugate brought to the viral surface by the high affinity of the ZA moiety for NA. Under irradiation of room light, the porphyrin component of conjugate **46** brings about reactive singlet oxygen to kill the attached viruses without damaging other healthy host cells. In contrast, a similar concentration of free porphyrin alone or in combination with zanamivir cannot accumulate to a high local concentration on the viral surface, and thus the destruction of influenza virus by light irradiation is ineffective.

In another aspect, the tetrameric ZA conjugate **46** can also take advantage of multivalent effect [118–121] to enhance the binding with influenza NA, which exists as a homotetramer on the surface of the virus, thus inducing aggregation of viral particles for physical reduction of the infectivity. Di-, tri-, tetra- and polyvalent ZA conjugates are also designed to increase the binding affinity with NA [122–128]. Klibanov and coworkers [129] implanted ZA and sialic acid molecules on the poly(isobutylene-*alt*-maleic anhydride) backbone for concurrent bindings with viral NAs and HAs, thus greatly increasing the anti-influenza activity by more than 1000 fold.

#### Conjugating zanamivir with caffeic acid to alleviate inflammation

**Fig. 15:**
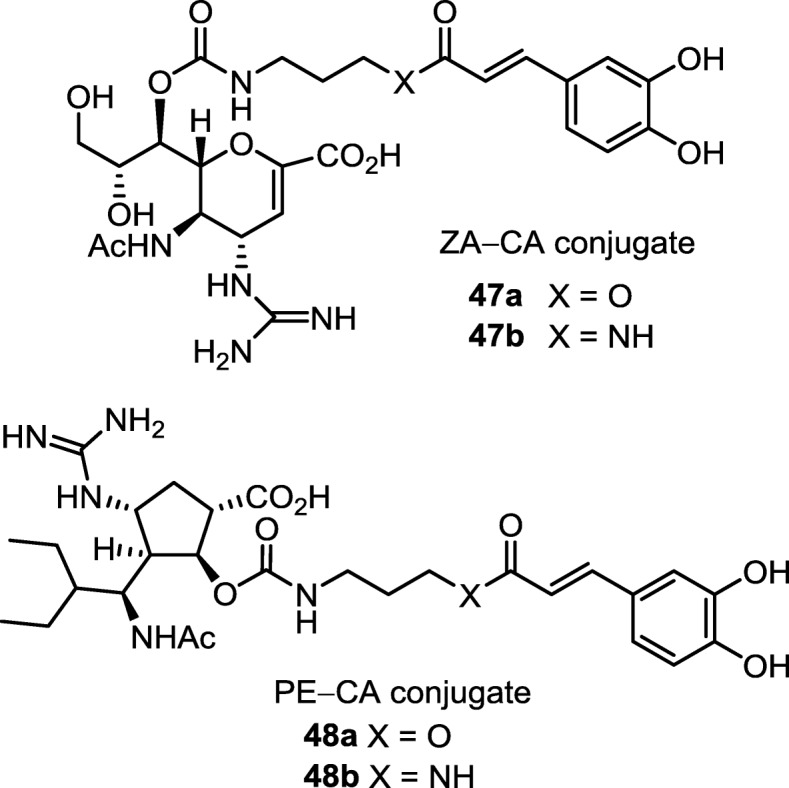
Enhanced anti-influenza activity of ZA−caffeate and PE−caffeate conjugates by synergistic inhibition of neuraminidase and suppression of the virus-induced cytokines

Influenza infection may induce uncontrolled cytokine storms as that happened in 2003 avian flu, resulting in the cross-species transmission of H5N1 avian virus to humans to claim a large number of lives. Since extension from the C_7_-OH would not interfere with NA binding, the dual functional ZA–caffeate conjugates **47a** and **47b** (Fig. 15) are prepared by connection of caffeic acid to ZA with ester or amide linkage [130]. The cell-based assays indicate that conjugates **47a** and **47b** effectively inactivate H1N1 and H5N1 influenza viruses with EC_50_ in nanomolar range. These conjugates also significantly inhibit proinflammatory cytokines, such as interleukin-6 (IL-6) and interferon-gamma (INF-γ), compared to ZA alone or in the presence of caffeic acid (CA).

Treatment with the ZA conjugates **47a** and **47b** improves the survival of mice infected with influenza virus. For example, treatment of conjugates **47a** and **47b** at 1.2 μmol/kg/day, i.e. the human equivalent dose, provides 100% protection of mice from lethal-dose challenge of influenza H1N1 or H5N1 viruses in the 14-day experimental period. Even at a low dose of 0.12 μmol/kg/day, conjugates **47a** and **47b** still significantly protect the H1N1 virus-infected mice, showing greater than 50% survival on day 14. ZA alone or anti-inflammatory agent alone cannot reach such high efficacy for influenza therapy [131, 132]. Although the combination of an NA inhibitor with anti-inflammatory agents is effective in treating influenza-infected mice [133, 134], the drug development may encounter problems with complex pharmacokinetics behavior. On the other hand, conjugates **47a** and **47b** bear ZA component for specific binding to influenza virus, thus delivering the anti-inflammatory component for in situ action to suppress the virus-induced cytokines. By incorporating a caffeate component, conjugates **47a** and **47b** also have higher lipophilicity to improve the pharmacokinetic properties.

#### Conjugating peramivir with caffeic acid as enhanced oral anti-influenza drug

The C_2_-OH group, which does not directly interact with NA protein [135, 136], is used for conjugation of peramivir with caffeic acid. The PE–caffeate conjugates **48a** and **48b** (Fig. 15) are nanomolar inhibitors against wild-type and mutated H1N1 viruses [137]. The molecular modeling of conjugate **48b** reveals that the caffeate moiety is preferably located in the 295-cavity of H275Y neuraminidase, thus providing additional interactions to compensate for the peramivir moiety, which has reduced binding affinity to H275Y mutant caused by Glu276 dislocation. By incorporating a caffeate moiety, conjugates **48a** and **48b** also have higher lipophilicity than PE. Thus, conjugates **48a** and **48b** provide better effect in protecting MDCK cells from infection of H275Y virus at low EC_50_ (~ 17 nM). Administration of conjugates **48a** or **48b** by oral gavage is effective in treating mice infected by a lethal dose of wild-type or H275Y influenza virus. In view of drug metabolism, since the ester bond in the conjugate **48a** is easily hydrolyzed in plasma, the conjugate **48b** having a robust amide bond may be a better candidate for development into oral drug that is also active against mutant viruses.

## Conclusion

In this review, the anti-influenza drugs are discussed with an emphasis on those targeting the NA glycoprotein. In order to generate more potent NA inhibitors and counter the surge of resistance caused by natural mutations, the structures of on-market anti-influenza drugs are used as templates for design of new NA inhibitors. In particular, we highlight the modifications of these anti-influenza drugs by replacing the carboxylate group in oseltamivir, zanamivir and peramivir with bioisosteres (e.g. phosphonate and sulfonate) to attain higher binding strength with influenza NA. The carboxylic acid can also be converted to ester prodrugs for better lipophilicity and bioavailability. Using lipophilic acyl derivatives of guanidine as prodrug of zanamivir and guanidino-oseltamivir can mitigate the problem of low bioavailability. The C_7_-OH in zanamivir and C_2_-OH in peramivir, which point outward from the active site of influenza NA, are suitable for derivatization. Conjugating zanamivir molecules to porphyrin not only enhances the NA inhibitory activity, but also effectively activates molecular oxygen to kill influenza viruses. The ZA–caffeate and PE–caffeate conjugates render higher efficacy than their parental compounds (ZA or PE) in treatments of the mice infected with human or avian influenza viruses. Using congeners and conjugates is a viable strategy to develop orally available anti-influenza drug that is also active to mutant viruses. Interdisciplinary collaboration is essential in development of new anti-influenza drugs, and synthetic chemists play an important role to reach the goal.

## Data Availability

Not applicable.

## Abbreviations

Boc: *tert*-butoxycarbonyl
CA: caffeic acid
DANA: didehydro-2-deoxy-*N*-acetylneuraminic acid
d-GlcNAc: *N*-acetyl-d-glucosamine
GOC: guanidino-oseltamivir carboxylate
HA: hemagglutinin
HNAP: 1-hydroxy-2-naphthoic acid
HWE: Horner−Wadsworth−Emmons
log *D*: distribution coefficient
log *P*: partition coefficient
MUNANA: 2-(4-methylumbelliferyl)-α-d-*N*-acetylneuraminic acid
NA: neuraminidase
NBS: *N*-bromosuccinimide
Neu5Ac: sialic acid
OC: oseltamivir carboxylate
OS: oseltamivir
PDT: photodynamic therapy
PE: peramivir
PP: peraphosphor
PP1Et: peraphosphor monoethyl ester
py: pyridine
RNP: ribonucleoprotein
TMS: trimethylsilyl
TMSBr: bromotrimethylsilane
TMSOTf: trimethylsilyl trifluoromethanesulfonate
TP: tamiphosphor
TP1Et: tamiphosphor monoethyl ester
TPG: guanidino tamiphosphor
TPG1Et: guanidino tamiphosphor monoethyl ester
ZA: zanamivir
ZP: zanaphosphor
